# Continuous Glucose Monitoring in Adolescents With Obesity: Monitoring of Glucose Profiles, Glycemic Excursions, and Adherence to Time Restricted Eating Programs

**DOI:** 10.3389/fendo.2022.841838

**Published:** 2022-02-25

**Authors:** Monica N. Naguib, Elizabeth Hegedus, Jennifer K. Raymond, Michael I. Goran, Sarah-Jeanne Salvy, Choo Phei Wee, Ramon Durazo-Arvizu, Lilith Moss, Alaina P. Vidmar

**Affiliations:** ^1^ Department of Pediatrics, Center for Endocrinology, Diabetes, and Metabolism, Children’s Hospital Los Angeles, Los Angeles, CA, United States; ^2^ Department of Pediatrics, The Saban Research Institute, Children’s Hospital Los Angeles, Los Angeles, CA, United States; ^3^ Department of Medicine, Research Center for Health Equity Samuel Oschin Comprehensive Cancer Institute, Cedars-Sinai Medical Center, Los Angeles, CA, United States; ^4^ Department of Preventive Medicine, Southern California Clinical and Translational Science Institute, Keck School of Medicine, Los Angeles, CA, United States; ^5^ Southern California Clinical and Translational Science Institute Biostatistics Core, The Saban Research Institute, Children’s Hospital Los Angeles, Los Angeles, CA, United States

**Keywords:** continuous glucose monitor (CGM), obesity, adolescent, time restricted eating, glycemic profile, adherence - compliance - persistence, glycemic excursion

## Abstract

**Background:**

Randomized controlled trials of time restricted eating (TRE) in adults have demonstrated improvements in glucose variability as captured by continuous glucose monitors (CGM). However, little is known about the feasibility of CGM use in TRE interventions in adolescents, or the expected changes in glycemic profiles in response to changes in meal-timing. As part of a pilot trial of TRE in adolescents with obesity, this study aimed to 1) assess the feasibility of CGM use, 2) describe baseline glycemic profiles in adolescents with obesity, without diabetes, and 3) compare the difference between glycemic profiles in groups practicing TRE versus control.

**Methods:**

This study leverages data from a 12-week pilot trial (ClinicalTrials.gov Identifier: NCT03954223) of late TRE in adolescents with obesity compared to a prolonged eating window. Feasibility of CGM use was assessed by monitoring 1) the percent wear time of the CGM and 2) responses to satisfaction questionnaires. A computation of summary measures of all glycemic data prior to randomization was done using EasyGV and R. Repeat measures analysis was conducted to assess the change in glycemic variability over time between groups. Review of CGM tracings during periods of 24-hour dietary recall was utilized to describe glycemic excursions.

**Results:**

Fifty participants were enrolled in the study and 43 had CGM and dietary recall data available (16.4 + 1.3 years, 64% female, 64% Hispanic, 74% public insurance). There was high adherence to daily CGM wear (96.4%) without negative impacts on daily functioning. There was no significant change in the glycemic variability as measured by standard deviation, mean amplitude glycemic excursion, and glucose area under the curve over the study period between groups.

**Conclusions:**

CGM use appears to be a feasible and acceptable tool to monitor glycemic profiles in adolescents with obesity and may be a helpful strategy to confirm TRE dosage by capturing glycemic excursions compared to self-reported meal timing. There was no effect of TRE on glucose profiles in this study. Further research is needed to investigate how TRE impacts glycemic variability in this age group and to explore if timing of eating window effects these findings.

## 1 Introduction

Continuous glucose monitoring (CGM) provides serial interstitial glucose measurements in a noninvasive manner through a wearable device called a continuous glucose monitor (1–3). An advantage of continuous glucose monitoring is to have real time information about glycemic excursions that can be aligned with activities of daily living to support glycemic control among individuals living with diabetes (2–4). Various CGM are approved by the U.S Food and Drug Administration (FDA) for use in youth with diabetes, however little is known about the role and utility of CGM in the management of youth with obesity without diabetes (5). Continuous glucose monitoring is increasingly used in obesity research in adults. Through these studies, the CGM has emerged as a useful tool to understand and monitor glycemic variability, occurrence of hypoglycemia, and overall efficacy of dietary interventions on glycemic excursions (5–7). Continuous glucose monitoring provides more specific glycemic information than does a single hemoglobin A1c value and is less burdensome than checking glucose by fingerstick or by an oral glucose tolerance test. A recent scoping review, conducted by this study group, explored the use of CGM in obesity-related research. While there was a wide variety of CGM uses and outcome metrics in adults, there were very few studies conducted in pediatric populations (5). Only eight studies in children and adolescents were identified, and all were relatively small (n = 17-118) and of very short duration (range of CGM wear = one to eight days) (3–8). When considering the use of CGM in adolescents without diabetes, many factors must be considered including the feasibility and acceptability of daily wear and the selection of metrics extracted from CGM.

Recently, several adult studies have utilized CGM with the implementation of time restricted eating (TRE) interventions as a mechanism to monitor 24-hour glycemic profiles as they relate to meal timing (9–12). Given that TRE consists of limiting daily food intake to a 10-hour period or less, followed by a daily fast of at least 14 hours with or without calorie restriction, 24-hour glucose surveillance is a useful tool to explore the impact of fasting periods on glycemic profiles over the course of the day. Some TRE studies in adults have demonstrated improvement in glycemic measures such as mean glucose, fasting glucose, and post-prandial glucose compared to those eating over prolonged time windows (9–12), however others have found no TRE treatment effect on glycemic variables (13–17). It has been hypothesized that this difference is most likely related to the time of day in which the eating period occurs, however further investigation is warranted to determine the exact mechanism of these inconsistencies. The majority of these studies used CGM to capture changes in glucose and to assess glycemic variability in response to implementation of TRE regimens over time. Outside of this study group, there is limited data on the use of TRE and CGM in adolescents with obesity (18, 19). TRE may be more sustainable and effective for adolescents because it removes the need for intensive counting of daily caloric intake and focuses on a straightforward task of consuming food during a pre-specified time window. In this age group, continuous glucose monitoring may be a useful tool to combine with TRE implementation, not only to assess glycemic variability overtime and in response to the intervention, but also to evaluate glycemic excursions in relation to meal timing to confirm intervention adherence in real time.

This present study leverages data from a randomized controlled pilot trial investigating the feasibility, acceptability, safety, and preliminary efficacy of TRE and CGM use in adolescents with obesity seeking treatment for weight management (19, 20). The aim of this study is to: 1) determine if adolescents with obesity without diabetes would be willing and able to wear a CGM daily for 12 weeks, 2) describe the glycemic profiles and variability obtained from CGM at baseline, and 3)describe the glycemic profiles and variability in groups practicing TRE versus control. We hypothesize that CGM use will be feasible, acceptable, and safe in this cohort, that glycemic profiles among the TRE groups would reflect decreased glucose variability, and that glycemic excursions would be a useful adjunct to other measures of dietary intake monitoring adherence to prescribed eating window.

## 2 Methods

### 2.1 Participants

Data was extracted from 50 adolescents (ages 14-18) with a body mass index (BMI) ≥95th percentile who were enrolled in a three-arm pilot trial testing the feasibility, safety, and preliminary efficacy of 8-hour TRE compared to a 12-hour control group (19, 20). Exclusion criteria included: 1) previous diagnosis of Prader Willi syndrome, brain tumor, or hypothalamic obesity; 2) serious developmental or intellectual disability, or previously diagnosed eating disorder; 3) inability to participate in the assessments (e.g. inability to wear a CGM); 4) previous bariatric surgery; 5) current use of medication that impacts weight or executive functioning (e.g., antipsychotics, sedatives, hypnotics, off-label obesity medication, insulin); 6) current participation in psychotherapy regarding weight or eating behavior; or 7) current participation in other interventional studies or clinical treatment related to weight management (20). Adolescents were randomized 1:1:1 *via* blocked randomization to be assigned to one of three intervention arms: 1) Arm 1: 12-hour eating window + blinded CGM (control schedule); (2) Arm 2: TRE over an 8-hour eating window with a 16-hour fasting window + blinded CGM; and (3) Arm 3: TRE over an 8-hour eating window with a 16-hour fasting window + CGM with real-time feedback (meaning the glucose data is viewable to the participant). Study procedures were approved by the Children’s Hospital Los Angeles (CHLA) Institutional Review Board. The study was reported according to the Consolidated Standards of Reporting Trials (CONSORT) guidelines and is registered with ClinicalTrials.gov (NCT03500835). Written informed consent was obtained from the adolescent and one parent or guardian.

### 2.2 Intervention

The specific details of the TRE intervention have been described previously (19, 20). In brief, all participants received a two-hour nutrition education session and were randomized to one of three groups as described above. Participants in the TRE groups were instructed to consume all their food in a self-selected eight-hour time window five days per week. They were required to set the time window at the consent visit. Participants reported their eating windows to the study team weekly. All participants wore Dexcom G6 continuous glucose monitors (CGM, Dexcom, San Diego, CA, USA) continuously for 13 weeks (week 0 to week 12 study period). All participants wore their CGM for 7-10 days (run-in period) prior to being randomized to capture baseline glycemic profiles. Participants were provided with a transmitter and enough sensors to replace the sensor every ten days. The participants and guardians were educated on how to use the CGM and received one-on-one coaching on how to change the sensor, which was completed either independently or under study team guidance. No glucometer calibration was required. Hypoglycemia alarm was set at 55 mg/dL and hyperglycemia alarm was set at 400 mg/dL. Study participants were encouraged to contact the study team for further instruction if any alarms sounded. At each weekly phone meeting, study staff monitored any adverse events and challenges related to CGM wear, including participant discomfort, skin adherence, and other issues. Participants and guardians were compensated a combined total of $200 in the form of gift card credit for their time spent participating in the study.

### 2.3 Measurements

At the start of the study, participants and guardians completed questionnaires regarding demographic information, past medical history, height, and weight. Participants received a wireless, Bluetooth scale upon consent. Participants’ height and weight measurements were collected by the participant and parent/guardian at home with the research coordinator monitoring the measurement collection *via* a HIPAA compliant virtual platform at weeks 1, 4, 8, and 12. BMI was calculated as kilograms per meter squared; BMI z-score (zBMI) and excess percent of the 95th percentile (%BMIp95) were determined utilizing the CDC growth charts.

#### 2.3.1 Feasibility of CGM Wear

Feasibility was determined by assessing the number of days that participants wore their CGM out of the prescribed time, and by responses to a standardized satisfaction questionnaire adapted from the CGM satisfaction scale (21) administered during weeks 4, 8, and 12 of the study. Adolescents were instructed to wear their CGM daily for the duration of the study and to report deviations from the protocol during weekly phone calls with the research staff. The percent of days that the CGM was worn was recorded by the Dexcom Clarity platform.

#### 2.3.2 Baseline Glycemic Profiles as Assessed From CGM Data

CGM data were reviewed at each study visit by the study team and downloaded after completion of the study. The CGM provided glucose levels every five minutes from the seven days prior to the intervention commencement until the final day of week 12 of the study. These data were utilized to compute the following measures using glycemic variability calculator software, easyGV (easyGV, University of Oxford, Oxford, England): mean, median, maximum, and minimum glucose levels, percent CGM wear , Glucose Management Indicator (GMI), standard deviation (SD), coefficient of variation (CV), daytime and nighttime mean glucose, daytime and nighttime glucose SDs, area under the curve (AUC), mean amplitude of glycemic excursion (MAGE), J index, continuous overlapping net glycemic action (CONGA), mean of daily difference (MODD), low blood glucose index, and high blood glucose index (1, 4, 6, 8, 22, 23). MAGE is a preferred measure of short-term within-day glycemic variability in clinical studies (22). CV is a primary measure of glycemic variability though SD is a more clinically familiar surrogate for CV. AUC is a recommended measure for research purposes to calculate the degree of hypoglycemia or hyperglycemia, and their associated duration (23). To assess changes in glycemic variability, CGM data was continuously collected over the study period and analyzed over time and between intervention arms.

#### 2.3.3 Glycemic Excursion Monitoring

##### 2.3.3.1 Nutrient Data System Recall (NDSR) 24 Hour Dietary Recall

The Minnesota Nutrition Data System for Research (2020 version) software was used to record and compute nutrient intake for all participants at weeks 0, 4, and 12 of the study (24, 25). Dietary recalls were obtained using the US Department of Agriculture Automated Multiple-Pass Method (AMPM). During each of the nutrition recall sessions, nutrient intake from the prior two days were recorded, with a total of six days of nutrient intake recorded throughout the study. For the participants in the TRE groups, efforts were made to obtain a dietary recall on a TRE and non-TRE day. The eating window was determined by recording the time of the first and last recalled caloric intake of the selected day between 12:00 am and 11:59 pm.

##### 2.3.3.2 Self-Report of Eating and Fasting Window

All participants were required to log and report their daily eating, which refers to the number of hours in the day during which calories were consumed. This information was shared with the study team at the weekly phone meetings and were logged into REDCap.

##### 2.3.3.3 Monitoring Glycemic Excursion

To explore the association between fasting and non-fasting and glycemic excursions, data from the Dexcom Clarity platform was reviewed and compared to dietary recall data recorded on NDSR. During the run-in period, all participants wore their CGM for at least seven days prior to randomization. The greatest glycemic excursion (excursion = the difference between minimum and maximum glucose reported on CGM tracing) and its associated minimum and maximum glucose were recorded for each participant daily during the run-in period, and during fasting and non-fasting periods during days that NDSR dietary recall was done during weeks 4 and 12. At weeks 4 and 12, the study staff identified the greatest glycemic excursion on the CGM tracing that occurred during the 48-hours of dietary recall collected during fasting and non-fasting periods. The fasting period was considered (from 3:00 AM to the first reported caloric intake, and from three hours after the last reported caloric intake until 11:59 PM). The non-fasting period was considered from the first reported caloric intake of the day until three hours after the last reported caloric intake.

### 2.4 Statistical Analysis

The distribution of all variables was examined prior to analysis. Descriptive statistics reported include median, minimum, and maximum for continuous variables, and frequency and percent for categorical variables. Continuous variables with normal distribution were summarized in mean and standard deviation, while median and range were utilized for variables with non-normal distribution. Categorical variables were summarized in frequency and percentage. Analysis of variance (ANOVA), Fisher’s Exact, and Kruskal-Wallis test were used to assess the differences in distribution of demographic characteristics and baseline anthropometrics across the three intervention groups. The computation of summary measures of glycemic data obtained from the CGM was done by an R package, CGM analysis. The change in CGM variables over time and the differential of intervention groups were assessed by univariate median quantile regression model with clustered standard errors to appropriately adjust for repeated measures. These results were described in beta estimate, β, with its associated 95% confidence interval and p-value. The change in CGM variables over time controlling for intervention arms was assessed by including interaction terms in the quantile regression model. Multivariate linear regression was used to assess the difference between fasting and non-fasting excursions between intervention arms at week 4 and 12. Results are presented as the Pearson Correlation coefficient, stratified by intervention arm regardless of the statistical significance of the interaction term. The relationship between fasting and non-fasting excursions and percent weight change was assessed using linear regression. In addition, multivariable linear regression was used to assess the differences in this relationship between intervention arms by including a time by independent variable (percent weight change, %BMIp95, and BMI z-score) interaction term. Pearson’s correlation coefficient was estimated and corresponding p-value for testing a difference from zero was calculated. Multivariable mixed-effects linear models were used to test different patterns of fasting and non-fasting excursions, as well as differences in time-patterns across intervention arms. Besides glycemic summary measures, all statistical computations were done in Stata/SE 17 (StataCorp, College Station, TX).

## 3 Results

### 3.1 Participant Characteristics

Descriptive statistics and baseline demographics are presented in **Table 1**. Fifty participants were consented for the study. Five participants withdrew from the study: two participants developed type 2 diabetes, two participants were unable to commit time to the study, and one participant withdrew due to personal family issues. Forty-three adolescents had sufficient CGM data and dietary recall data to analyze. There was no significant difference in the baseline characteristics between the participants who did and did not complete the study (all p values >0.05). Participants had a median age of 16.4 years (range 14-18 years), were predominantly Hispanic (64%), publicly insured (74%), with a household income <$50,000 (70%), and female (72%) with a mean BMI z-score of 2.30 SD.

**Table 1:** Demographic characteristics and baseline anthropometrics.

	Total (n=50)	Arm 1:Control (n=15)	Arm 2:TRE + blinded CGM (n=19)	Arm 3:TRE + real-time CGM feedback (n=16)	*p*
**Age (in years)** ^1^	16.43 ± 1.17	16.38 ± 1.25	16.16 ± 1.16	16.80 ± 1.09	0.3^a^
**Sex** ^2^					0.8^b^
Male	14 (28.0)	3 (20.0)	6 (31.5)	5 (31.2)	
Female	36 (72.0)	12 (80.0)	13 (68.4)	11 (68.7)	
**Race** ^2^					0.05^b^
White	5 (10.0)	3 (20.0)	1 (5.2)	1 (6.0)	
Black	3 (6.0)	1 (6.6)	2 (10.5)	0 (0)	
Asian	4 (8.0)	3 (20.0)	1 (5.2)	0 (0)	
Hispanic	27 (54.0)	7 (46.7)	13 (68.4)	7 (43.8)	
Am. Indian	2 (4.0)	0 (0)	1 (5.2)	1 (6.2)	
Mixed race	6 (12.0)	1 (6.6)	0 (0)	5 (31.2)	
**Ethnicity** ^2^					0.1^b^
Non-Hispanic	15 (30.0)	8 (53.3)	4 (21.1)	3 (18.7)	
Hispanic	32 (64.0)	7 (46.6)	14 (73.6)	11 (68.7)	
**Weight (kg)** ^3^	101.4 (87.9, 123.8)	104.3 (74.8, 123.1)	99.5 (84.6, 123.2)	110.5 (92.2, 128.3)	0.9^c^
**%BMIp95** ^3^	125.9 (111, 158)	141.1 (114.4, 167.0)	122.6 (110.0, 158.5)	123.9 (109.8, 159.1)	0.9^c^
**BMI z-score** ^1^	2.30 ± 0.5	2.34 ± 0.5	2.28 ± 0.4	2.30 ± 0.5	0.9^a^

^a^Analysis of variance; ^b^Fisher’s Exact test; ^c^Analysis of variance in log scale.

^1^Mean ± standard deviation; ^2^Frequency (percentage); ^3^Median (interquartile range).

### 3.2 CGM Feasibility, Safety, and Acceptability

**Figure 1:**
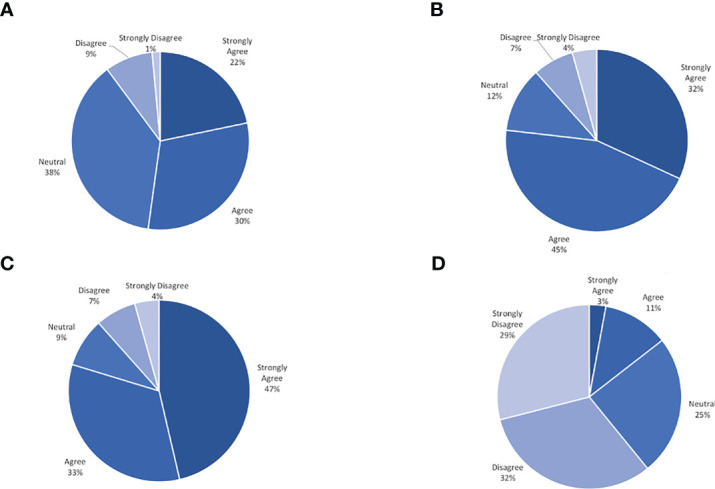
Continous glucose monitor satisfaction survey result. **(A)** Helps me feel more satisfied with my weight management. **(B)** Gives me information about my glucose that is useful. **(C)** Helps me identify how food and activity affect me. **(D)** Makes me feel more frustrated about my weight.

On average all participants wore their CGM 96.4% (30-100%) of the prescribed wear time across the three groups. All 12 weekly phone calls and all follow-up surveys and clinical data were collected from 86% of participants. No significant safety events with respect to wearing the CGM daily were identified. Few barriers to participation were reported by adolescents. The most common barriers pertained to wearing the CGM. On a 5-point Likert scale from 1 = “strongly disagree” to 5 = “strongly agree”, participants rated the CGM as causing “too much skin irritation” as 2.2 and rated the CGM as “too painful” to use as 1.7. Eight participants (16%) reported skin irritation at least once during the twelve weeks of the study, and ten participants (20%) reported mild bleeding at the insertion site at any time point in the study. Of those reporting skin irritation, only two participants stopped using the CGM for the remainder of the study. Another commonly reported barrier elicited during the weekly phone calls was the CGM falling off due to poor adhesion or due to other mechanisms (34%). One participant reported feeling uncomfortable wearing the CGM due to its use in patients with diabetes, and felt that by wearing it, there was something ‘wrong’ with her (this patient later withdrew from the study). Several participants reported their sensor alarming as low while sleeping, however with repositioning this self-resolved, consistent with a false low due to increased pressure on sensor site. In general, adolescents reported favorable experiences with utilizing a CGM. On a 5-point Likert scale from 1 = “strongly disagree” to 5 = “strongly agree”, participants rated wearing CGM as helping them feel more satisfied with their weight management as 3.9 and wearing CGM as helping them identify how food and activity affect them as 4.1. Based on adolescents’ responses to the satisfaction survey, the CGM was viewed favorably. An overview of the satisfaction survey results is displayed in **Figure 1**. Ninety-five percent of adolescents wore their CGM for the total duration of the study period. More than 88% of the entire cohort commented on positive experiences with the CGM.

### 3.3 Glycemic Profiles

Given this study was conducted during the COVID-19 pandemic, restrictions on clinical research prevented the study team from collecting laboratory-confirmed markers of beta cell function (i.e., serum fasting glucose, hemoglobin A1c, insulin level, etc.) as planned. All data reported was extracted from the CGM tracings (**Tables 2A, B**). At baseline, the mean random glucose across the three groups was 108.9 mg/dL (SD 16.8 mg/dL), with a GMI of 5.4% (SD 0.1%), and with no significant difference over time or across intervention groups (all p>0.05). **Table 2A** outlines the glycemic variables at baseline, week 4, and week 12 by intervention arm. **Table 2B** displays the calculated glycemic variability metrics at baseline, week 4, and week 12 by intervention arm.

**Table 2a:** Continuous glucose monitor variables.

CGM variable	Arm 1: Control Group (N=16)				Arm 2: TRE + blinded CGM (N=16)				Arm 3: TRE + real-time CGM feedback (N=15)			
	Week 0	Week 4	Week 8	Week 12	Week 0	Week 4	Week 8	Week 12	Week 0	Week 4	Week 8	Week 12
**% CGM wear**	97 [41-100]	98 [54-100]	99 [85-100]	100 [80-100]	97.5 [64-109]	89.5 [48-100]	96 [46-100]	100 [39-100]	84.5 [27-100]	98 [30-100]	97.5 [31-100]	100 [78-100]
**Mean glucose**	113 [86.8-148.8]	109.2 [93.8-134.9]	114.3 [92.6-133.1]	105.9 [87.9-128.3]	106.6 [91.3-146.6]	108.1 [89.2-169.6]	109.7 [88.4-153.1]	118.9 [91-144.9]	107 [91-215.2]	108.5 [93.2-160.7]	121.2 [104.4-145.1]	104.9 [96-132]
**Median glucose**	109 [86-136]	106 [91-129]	112 [91-131]	102.5 [86-127]	104.5 [89-135]	105.5 [88-160]	107 [86-137]	116 [89-133]	103 [88-201]	106 [91-148]	112.5 [102-137]	102 [96-133.5]
**GMI**	5.6 [4.7-6.8]	5.4 [4.9-6.3]	5.6 [4.9-6.3]	5.3 [4.7-6.1]	5.4 [4.8-6.7]	5.4 [4.7-7.5]	5.5 [4.7-7]	5.8 [4.8-6.7]	5.4 [4.8-9.1]	5.4 [4.9-7.2]	5.9 [5.3-6.7]	5.3 [5-6.2]
**SD glucose**	17.5 [13.2-40.9]	18.4 [12.4-26.1]	19.1 [10.9-50.7]	14.1 [9.4-28.2]	15.7 [11.4-37.9]	16.7 [9.6-46.8]	16.6 [10.6-52.9]	19.2 [8.7-41.8]	17.2 [7.8-65.4]	18.1 [13.2-46.2]	25.4 [15.7-36.9]	17.3 [7.1-23.7]
**CV glucose**	0.2 [0.1-0.3]	0.2 [0.1-0.2]	0.2 [0.1-0.4]	0.1 [0.1-0.3]	0.2 [0.1-0.3]	0.2 [0.1-0.3]	0.2 [0.1-0.4]	0.2 [0.1-0.3]	0.2 [0.1-0.3]	0.2 [0.1-0.3]	0.2 [0.2-0.3]	0.2 [0.1-0.2]
**Minimum glucose**	66 [40-80]	67 [40-97]	46 [40-86]	67 [41-100]	63 [40-90]	68.5 [40-93]	53 [40-84]	75 [40-106]	71 [42-104]	58 [40-83]	63 [51-82]	65 [62-111]
**Maximum glucose**	181.5 [146-274]	179.5 [145-231]	177 [156-225]	159 [102-193]	179 [144-256]	180 [121-308]	165 [133-377]	169 [114-284]	179.5 [134-500]	171 [143-321]	213.5 [166-272]	181 [118-208]
**Daytime mean glucose**	111.1 [84.9-139.5]	109.6 [91.7-136.2]	115.8 [94.7-136]	103.6 [84.4-125.8]	106.7 [90.9-144]	107.9 [89.5-170.3]	109.7 [86.6-149.6]	113 [84.1-149]	106.4 [91.5-206.8]	107.8 [92.2-156.2]	124.1 [105.4-149.1]	106.6 [95.4-127.7]
**Daytime SD**	17.1 [12.9-37.2]	18.2 [10.8-27.6]	19.7 [10.3-53.5]	13.2 [9.1-23.4]	16.4 [11.5-36.1]	17.2 [9.5-42.2]	16.7 [11.2-54.1]	18.7 [8.2-45.2]	18 [8.4-69.9]	18.1 [13.2-48.4]	25.2 [17.1-40.1]	18.4 [6.9-25.5]
**Nighttime mean glucose**	112.7 [90.4-172.6]	104.8 [87.9-131.9]	112.4 [88-131.8]	109.2 [86.3-137.9]	104.9 [92.2-153.4]	107.9 [87.6-168.1]	111.1 [87.3-161.8]	121.7 [84.8-134.8]	107.6 [89.6-230.3]	108.8 [91.9-184.1]	114.1 [100.6-148.2]	100.6 [97.2-134]
**Nighttime SD**	16.6 [9.8-40.5]	15.2 [9.1-21.8]	13.6 [10.4-26.6]	13.6 [3.9-31.6]	14.3 [4-41.7]	15.4 [7.6-56.4]	13.3 [7.1-49]	15.6 [6.8-29.9]	14.9 [5.8-63.4]	15.7 [1.2-53.8]	18.2 [11.3-36.2]	14 [3.8-19.8]
**% time spent** **70-150 mg/dL**	99.2 [76.1-99]	99.0 [87.5-100]	99.0 [70.1-100]	99.6 [92.5-100]	97.8 [79.4-100]	98.4 [34.1-100]	98 [73.2-100]	98.6 [78-100]	97.2 [25-100]	97.6 [70.8-100]	93.5 [83.4-99.9]	98.5 [96.4-100]

%, Percent; SD, Standard deviation; GMI, Glucose management indicator; CV, Coefficient of variation.

*Analyses were conducted of the difference in each CGM variable overtime and across intervention arm and all p-values were >0.05.

**Table 2b:** Calculated glycemic variability metrics.

	Arm 1: Control Group (N=16)				Arm 2: TRE + blinded CGM (N=16)				Arm 3: TRE + real-time CGM feedback (N=15)			
	Week 0	Week 4	Week 8	Week 12	Week 0	Week 4	Week 8	Week 12	Week 0	Week 4	Week 8	Week 12
**Total AUC**	1082927.5 [383675.0-1371690.0]	1036276.3 [103755.0-1173880.0]	940360.0 [66137.5-1179470.0]	620237.5 [85490.0-1015287.5]	968666.3 [700672.5-1343130.0]	901236.3 [399642.5-1301797.5]	919707.5 [527730.0-1503500.0]	104640.0 [48470.0-1459500.0]	765670.0 [109612.5-1753510.0]	958930.0 [88550.0-1619167.5]	1020900.0 [264717.5-1186995.0]	907077.5 [69320.0-989517.5]
**MAGE**	33.2 [23.1-77.2]	33.8 [23.8-56.3]	35.9 [21.2-85.5]	28.9 [17.1-49.4]	31.2 [22.2-78.2]	31.7 [16.1-76.9]	33.4 [21.4-89.8]	35.7 [14.4-78.3]	33.0 [19.6-99.2]	36.4 [26.3-87.0]	46.3 [32.8-86.0]	36.4 [12.7-52.2]
**J index**	17.0 [10.0-36.0]	16.9 [12.0-25.9]	17.5 [11.7-29.5]	14.2 [9.6-21.6]	15.0 [11.3-34.1]	15.8 [9.8-46.8]	15.4 [9.8-42.5]	18.5 [9.9-34.9]	15.1 [11.4-71.0]	15.5 [11.5-42.8]	22.9 [14.4-33.0]	15.5 [11.0-19.3]
**CONGA**	16.4 [11.5-33.6]	17.5 [11.2-25.6]	17.6 [11.4-35.4]	15.2 [7.9-26.5]	15.4 [10.0-29.7]	15.7 [7.8-32.2]	16.1 [10.2-34.8]	20.7 [10.7-30.6]	16.8 [9.8-37.5]	16.9 [11.4-33.7]	23.8 [16.8-39.9]	17.8 [8.8-25.7]
**MODD**	15.4 [12.0-36.0]	18.2 [11.0-23.3]	16.8 [9.7-25.0]	14.5 [8.7-23.5]	15.9 [8.2-40.6]	16.4 [8.3-51.5]	14.5 [9.5-45.3]	31.3 [13.2-43.9]	17.7 [10.2-63.8]	17.2 [12.0-46.6]	25.6 [16.0-39.3]	14.9 [9.2-25.8]
**LBGI**	0.9 [0.3-3.4]	0.9 [0.1-3.5]	1.2 [0.4-11.9]	1.2 [0.1-3.6]	1.0 [0.2-2.9]	1.1 [0.2-2.7]	0.9 [0.3-3.1]	0.8 [0.0-6.6]	1.1 [0.1-3.5]	1.3 [0.3-2.5]	1.0 [0.5-1.5]	1.3 [0.0-1.5]
**HGBI**	1.0 [0.3-5.3]	0.9 [0.2-2.8]	0.8 [0.3-4.9]	0.6 [0.2-2.5]	0.8 [0.2-4.8]	0.9 [0.0-7.9]	0.8 [0.2-7.4]	1.2 [0.0-5.3]	0.9 [0.2-15.4]	0.8 [0.3-7.0]	2.4 [0.8-4.8]	1.0 [0.0-2.0]

AUC, Area under the curve; MAGE, Mean amplitude of glycemic excursion; CONGA, Continuous overlapping net glycemic action; MODD, Mean of daily difference; LBGI, Low blood glucose index; HBGI, High blood glucose index.

*Analyses were conducted of the difference in each CGM variable overtime and across intervention arm and all p-values were >0.05.

### 3.4 Glycemic Excursions

**Figure 2:**
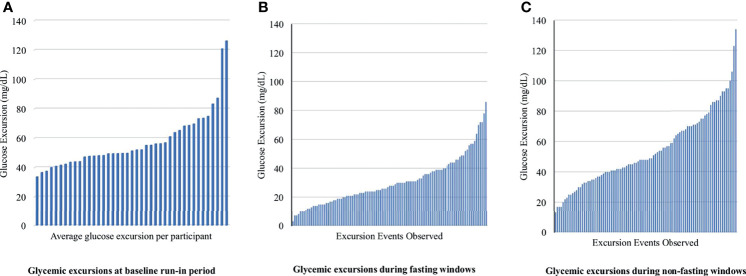
Fasting and non-fasting glycemic excursion events observed for each participant at baseline, week 4 and 12. Glycemic excursion is defined as the difference between the minimum and maximum glucose levels observed during the period of widest glycemic variability **(A)** displays the average glycemic excursion observed for each participant during the run-in period at baseline. **(B, C)** display glycemic excursion observed during fasting and non-fasting on days that 24-hour dietary recall was obtained. There were 105 unique glycemic excursion events extracted from 39 participant’s CGM data.

Available data (CGM plus dietary recall data) were obtained from 93% (40/43) of adolescents during the run-in period, 77% (33/43) of adolescents at the 4-week follow-up visit, and 53% (23/43) at the 12-week follow-up visit. At baseline, the mean glycemic excursion over a 24-hour period was 57.6 mg/dL (SD 19.7 mg/dL, range 8 to 174 mg/dL) (**Figure 2A**). At weeks 4 and 12, all participants with both dietary recall data and adequate CGM tracing were included (n = 39). During fasting periods, the mean glycemic excursion was 29.4 ± 13.7 mg/dL at week 4, and 30.1 ± 12.5 mg/dL at week 12 (range 3 to 86 mg/dL) (**Figure 2B**). During non-fasting periods, the mean glycemic excursion was 53.9 ± 20.8 mg/dL at week 4, and 56.3 ± 23.8 mg/dL at week 12 (range 13 to 134 mg/dL) (**Figure 2C**). Of note, three participants developed type 2 diabetes over the study course, confirmed with serum testing, and therefore their data was analyzed separately (fasting glycemic excursion range = 57 to 189 mg/dL).

### 3.5 Differences in Glycemic Excursions Between TRE and Prolonged Eating Window

**Figure 3:**
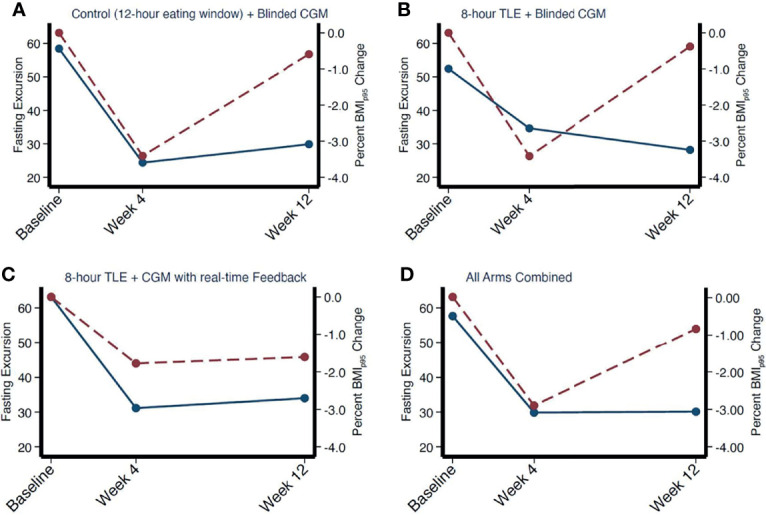
Correlation between mean fasting excursion and weight change over time by intervention arm **(A–C)** and combined **(D)**. Fasting excursion denoted by red dotted line. Weight change denoted by solid blue line.

Applying a univariate median quantile regression model with clustered standard errors to appropriately adjust for repeated measures with intervention arm as an interaction term there was no significant change in MAGE, SD, glucose AUC, or fasting or non-fasting glycemic excursions between intervention arms (all p>0.05). There was a greater decrease in fasting excursion noted from baseline to week 4 across all groups (with no between-group difference, all p values >0.05) with a stabilization between weeks 4 and 12. This trend paralleled the average weight status change noted over the study period. As shown in **Figure 3**, at week 4, there appeared to be a relationship between fasting glycemic excursions and weight status change, however on correlation analysis there was no significant association with change in weight (in kg, correlation coefficient = 0.19, p = 0.3, 95% CI [-0.17, 0.50]), %BMIp95 (correlation coefficient = 0.26, p = 0.15, 95% CI [-0.09, 0.55]), and BMI z-score (correlation coefficient = 0.06, p = 0.7, 95% CI [-0.29, 0.40]). There was no significant relationship between changes in glycemic excursion during fasting and weight status at week 12 (all p>0.05).

## 4 Discussion

To our knowledge, this is the first study to simultaneously examine the feasibility of continuous glucose monitoring in adolescents with obesity participating in a TRE intervention, describe baseline glycemic profiles in adolescents with obesity, without diabetes, and evaluate the impact of TRE on glycemic variability compared to a prolonged eating window. Consistent with our hypothesis, CGM use was found to be feasible, acceptable, and well-tolerated in this cohort. Secondly, although TRE was well-tolerated and adhered to [as previously reported by this study group (19)], there was no significant difference in glycemic variability computed from 13 weeks of CGM data between adolescents participating in TRE compared to those eating over a prolonged time window. Finally, CGM data may be a novel tool to monitor adherence to TRE by comparing glycemic excursions to dietary recall data.

Despite clinical concern that youth with obesity without diabetes would not wear a CGM, the adherence was very high, even relative to adolescents with type 1 diabetes (26). Barriers to CGM use that were reported, such as skin issues, are not unique to this cohort and have been reported with similar frequency by adolescents in the type 1 diabetes population (27). Adolescents reported that wearing the CGM promoted accountability and motivation for adherence to the prescribed dietary program. This highlights the importance of wearable technology as a research tool to capture biochemical data in real time, and as a behavioral tool in obesity research. Two recent systematic reviews have evaluated the use of wearable technology for weight loss in individuals with obesity and showed that wearable technology can be effective for weight loss and improvement in daily physical activity across the lifespan (28). Not only can CGM be utilized as a therapeutic intervention, but also as a method to capture 24-hour glycemic profiles in real time. This work adds to the growing discussion about the use of CGM in both clinical and research settings in those without diabetes. The present study was conducted during the COVID-19 pandemic and therefore traditional methods to evaluate beta-cell function and glycemic variability were limited. Continuous glucose monitoring provided a useful option to track remote data overtime and allow for this study to be conducted during a period in which research restrictions limited in-person assessment. Previous studies have also shown that glycemic data from CGM can be utilized to create “glucotypes”, or patterns of glycemic responses, that can predict conversion to prediabetes or type 2 diabetes with more specificity than traditional methods of diagnosis (28, 29).

The use of CGM in TRE interventions provides a possible solution to one of the great challenges of implementing TRE studies which is the ability to accurately monitor intervention adherence over the study period. Standard methods to monitor adherence to dietary interventions include 24-hour dietary recall, food timing questionnaires, diet diaries or checklists, and picture-based applications (24, 25, 28). However, the validity of these instruments is suboptimal and subject to recall and response biases, lack of validation, and errors in reporting (24, 25). It has also been shown in both pediatric and adult populations that individuals with higher weight tend to underreport energy intake, creating a systematic bias that attenuates the association between caloric intake and body mass index (24). With the growing interest in TRE, there is a need to be able to confirm fasting windows across various cohorts to verify dosage of the intervention received. In the absence of diabetes, glycemic excursions during fasting are expected to be quite narrow among adolescents with obesity. By comparing dietary recall data with CGM tracings we were able to describe how glycemic excursions differ during periods of fasting and non-fasting in adolescents with obesity without diabetes. Provided that expected glycemic excursions in settings of fasting and non-fasting are well-classified, supplementing dietary recall with continuous glucose monitoring may allow for a more objective assessment of TRE dosage. Certainly, this method is not without limitations. First, there is heterogeneity in glycemic excursions captured on CGM tracing depending on baseline beta-cell responsiveness that should be accounted for. Second, glycemic excursions differ based on the type of macronutrients consumed and at what time of day. For example, a participate could consume a low carbohydrate-based meal and have minimal glucose variability noted on CGM. Third, the evaluation of the CGM tracing is still mapped onto the dietary recall as the starting place to evaluate for fasting versus non-fasting periods and is thus at risk for the biases discussed above, as the study team could not confirm all fasting and non-fasting periods beyond what was recorded in the dietary recall. Despite these limitations, the addition of continuous glucose monitoring may allow for improved scalability and efficiency of dietary adherence monitoring for large scale clinical trials of novel dietary interventions such as TRE.

Forth, in this cohort, there was no significant difference in glycemic profiles or glycemic variability between TRE and control groups over time. As discussed in detail by Vidmar et al. (19), this feasibility trial was not powered to evaluate efficacy of TRE on the secondary outcomes of weight loss and glycemic variability. Therefore, the lack of between group difference was most likely related to the small sample size, lack of difference in weight change between groups, and self-selected late eating window. There have been several recent studies published in adult cohorts suggesting that the impact of TRE on weight and glycemic variability is most affected by the time of day of the eating window (7, 30), with a preferential effect noted in early TRE compared to late TRE. In this cohort, all adolescents selected an afternoon/evening eating window in the TRE group which may have contributed to the null findings. More investigation is needed to determine how TRE impacts metabolic outcomes in adolescents with obesity without diabetes in larger, fully powered trials. Finally, this study was conducted during the height of the COVID-19 pandemic in which spontaneous and structured physical activity and food intake suffered considerable limitations which may have impacted the glycemic profiles in this cohort.

### 4.1 Limitations

Several study limitations should be acknowledged. First, given the small sample size, these findings are preliminary and may not generalize to different populations and settings. Second, any form of dietary recall is subject to recall bias, and thus may have affected interpretation of glycemic excursions. Thirdly, there was missing CGM data that could not be recorded either due to connectivity issues or due to participants not wearing the CGM (loss of CGM supplies, sensors falling off or expiring prior to the end of the study period), which was most pronounced by the week 12 visit. Finally, there were very low glucose levels in some participants because of pressure applied to the CGM that we were not able to deem as accurate in the data set. Cost analysis was not performed, so data on the cost-effectiveness of dietary recall coupled with CGM use compared to current adherence monitoring methods could not be analyzed.

## 5 Conclusions

In this study, continuous glucose monitoring was found to be a feasible way to capture real-time glycemic excursions over a 12-week period in adolescents participating in a TRE intervention. CGM tracings were utilized to catalog glycemic profiles during fasting and non-fasting periods in adolescents with obesity without diabetes. As expected, there was a significant difference in mean excursions noted during fasting and non-fasting over the study period. We propose the use of CGM data in combination with dietary recall and self-report of meal-timing as a possible method to track adherence to TRE in adolescents. Finally, although in this study there was no difference in glycemic variability between TRE and control, further, fully powered, large randomized controlled trials are required to determine the impact of TRE on glycemic variability in this age group.

## Data Availability Statement

The original contributions presented in the study are included in the article/supplementary material. Further inquiries can be directed to the corresponding author.

## Ethics Statement

The studies involving human participants were reviewed and approved by Children’s Hospital Los Angeles Institutional Review Board. Written informed consent to participate in this study was provided by the participants’ legal guardian/next of kin.

## Author Contributions

MN, EH, JR, MG, S-JS, CW, RD-A, LM, and AV conceptualized and designed the study, drafted the initial manuscript, and reviewed and revised the manuscript. All authors approved the final manuscript as submitted and agree to be accountable for all aspects of the work.

## Funding

This work was supported in part by grants (1) UL1TR001855 from the National Center for Advancing Translational Science (NCATS) of the U.S. National Institutes of Health, (2) NIH/NCRR SC-CTSI Grant Number UL1TR000130, (3) National Institute on Minority Health and Health Disparities (NIMHD) Obesity Health Disparities Research Center (U54MD000502; SS/Dutton), the Eunice Kennedy Shriver National Institute of Child Health and Human Development (NICHD. R01HD092483; de la Haye/SS), (4) the National Cancer Institute (NCI; R01CA258222, Figueredo/SS/Peterson), (5) The Saban Research Institute of Children’s Hospital Los Angeles, and (6) The Gustavus and Louise Pfeiffer Research Foundation TGA010553. Dexcom donated Dexcom G6 sensors, transmitters, and receivers for use in this study.

## Author Disclaimer

The content is solely the responsibility of the authors and does not necessarily represent the official views of the National Institutes of Health. 

## Conflict of Interest

The authors declare that the research was conducted in the absence of any commercial or financial relationships that could be construed as a potential conflict of interest.

## Publisher’s Note

All claims expressed in this article are solely those of the authors and do not necessarily represent those of their affiliated organizations, or those of the publisher, the editors and the reviewers. Any product that may be evaluated in this article, or claim that may be made by its manufacturer, is not guaranteed or endorsed by the publisher.
